# Deducing topology of protein-protein interaction networks from experimentally measured sub-networks

**DOI:** 10.1186/1471-2105-9-301

**Published:** 2008-07-03

**Authors:** Ling Yang, Thomas M Vondriska, Zhangang Han, W Robb MacLellan, James N Weiss, Zhilin Qu

**Affiliations:** 1 Departments of Medicine (Cardiology), David Geffen School of Medicine at the University of California, Los Angeles, California 90095, USA; 2 Physiology, David Geffen School of Medicine at the University of California, Los Angeles, California 90095, USA; 3 Anesthesiology, David Geffen School of Medicine at the University of California, Los Angeles, California 90095, USA

## Abstract

**Background:**

Protein-protein interaction networks are commonly sampled using yeast two hybrid approaches. However, whether topological information reaped from these experimentally-measured sub-networks can be extrapolated to complete protein-protein interaction networks is unclear.

**Results:**

By analyzing various experimental protein-protein interaction datasets, we found that they are not random samples of the parent networks. Based on the experimental bait-prey behaviors, our computer simulations show that these non-random sampling features may affect the topological information. We tested the hypothesis that a core sub-network exists within the experimentally sampled network that better maintains the topological characteristics of the parent protein-protein interaction network. We developed a method to filter the experimentally sampled network to result in a core sub-network that more accurately reflects the topology of the parent network. These findings have fundamental implications for large-scale protein interaction studies and for our understanding of the behavior of cellular networks.

**Conclusion:**

The topological information from experimental measured networks network *as is *may not be the correct source for topological information about the parent protein-protein interaction network. We define a core sub-network that more accurately reflects the topology of the parent network.

## Background

Biological systems are characterized by extremely complex interacting networks of nucleotides, proteins, metabolites and other molecules. It has become increasingly clear that to understand the function of a cell, one must understand the function of these networks. Because the topological characteristics of a network are believed to determine basic properties of its function [1-4], a primary goal in analyzing biological networksis to determine how the interacting elements (nodes) are connected toeach other (edges or links). The commonly used large-scaleexperimental approaches (yeast two hybrid and affinity pull-down combined with mass spectrometry) for mapping protein-protein interaction networks are extremely useful to sample portions of the entire network, however, they have well recognized limitations: (i) some interactions are missed (false negatives); (ii) spurious interactions are detected (false positives); (iii) interactions are assumed to be direct (binary analyses lose hierarchical information); and (iv) some proteins function better than others in a protein interaction assay [5,6]. "Sticky" proteins may be less likely to have false negatives, but it remains an empirical argument as to whether these proteins are also more likely to have false positives. Other factors contributing to these limitations include effects of affinity tag interactions, effects of antibody binding, influence of subcellular localization and protein activity, and post-translational modifications.

A general theoretical question is whether there is a way to sample a network so that the topological information of a sub-network can reflect well that of the original network. This issue was addressed by recent theoretical studies of Stumpf and colleagues [7,8] who showed that a randomly-sampled sub-network from an Erdös-Rényi random network is still an Erdös-Rényi random network; the same is true for an exponential network. When the original network is scale-free, however, the randomly sampled sub-network is not truly scale-free, but the degree distribution is still very close to a power-law. These findings suggest that a randomly-sampled sub-network may still largely maintain the topological information of the original scale-free network. Besides the maintenance of degree distribution, we also numerically analyzed the network motifs and found that the motif structures were also maintained after random sampling (Additional file 1 Fig.S1). Therefore, a practical question that arises is whether the sub-networks measured by the large-scale experimental approaches can be used to deduce topological information of the original networks. The answer to this question remains largely unclear. In a recent computational analysis [9], it was found that the power-law degree distributions of sampled networks reported in previous studies [3,4,10-13] may be a consequence of the manner in which the data are acquired and the low coverage of the complete (i.e., the "actual") protein-protein interaction networks. Besides the degree distribution and network motifs, other topological properties of the randomly sampled network, such as degree exponent, average path length and clustering coefficient, can be quite different from the original network when the size of sampled network is smaller than that of the original one [14,15]. Nevertheless, based on these previous studies [7-9] and our simulations (Additional file 1 Fig.S1), a sample that reflects the degree distribution and percentage of network motifs of the original network should: be randomly acquired and contain a high degree of coverage of the parent network. By analyzing several experimentally measured protein-protein interaction networks in the present study, we demonstrate that these experimental samples do not constitute random samples, likely due to the aforementioned experimental considerations. This observation highlights that the experimentally-measured sub-networks may not be the correct source for topological information about the parent protein-protein interaction network, raising the distinct possibility that previous analyses of biological networks [3,4,10-13,16-22] make inappropriate conclusions about topology. Although we conclude in this study that the current experiment datasets cannot be used directly for deducing topological information of the original network, we hypothesized that there is a *core sub-network *(CSN) within the experimentally sampled network that can better retain the topological information of the original protein-protein interaction network.

## Results

### Properties of experimentally-measured protein-protein interaction networks

**Figure 1:**
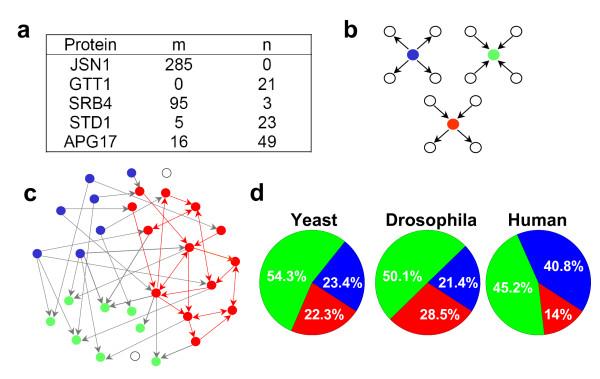
**Properties of measured protein-protein interaction networks**. a. Table showing example proteins with their respective links when tagged as baits or preys [23]. m is the total number of preys linked to the given protein when it is a bait; n is the total number of baits linked to the given protein when it is a prey. b. Three experimental behaviors of a protein in an interaction experiment. The blue protein acts only to detect other proteins (a 'pure bait') but itself is not a prey; the green protein acts only to be detected by other proteins (a 'pure prey'), itself not functioning as a bait; and the red protein acts as both bait and prey. c. Schematic plot of network structure of an experimentally measured network. d. Pie chart for the three types of proteins, color-coded as in Fig. 1b, for the experimental protein-protein interaction data from yeast [23], *Drosophila *[20], and human [12].

Despite the insights obtained by Stumpf and colleagues [7,8] regarding degree distribution and our numerical analyses of network motifs in randomly sampled networks (Additional file 1 Fig.S1), one is still faced with the problem that experimental sampling may not be random due to one or more of the following reasons: (i) some proteins are used as either bait or as prey, but not both; (ii) experimental results often contain data from different laboratories, species, techniques, etc.; and (iii) even if all proteins under analysis are used as both baits and preys (e.g., large scale yeast two-hybrid approaches), the relative ability of a protein to "behave as a bait" may not be equivalent to (and sometimes is completely different from) its ability to "behave as a prey" due to a variety of reasons. For example, the yeast protein-protein interaction network by Ito et al [23], all 6,000 proteins were used both as baits and preys, but in the resultant network, many proteins exhibited a preferential capacity to act as either a bait or a prey, while some do both. Figure 1a shows five example proteins from this dataset: JSN1 linked to 285 preys when it was used as a bait, but linked to no baits when it was used as a prey; in contrast, GTT1 linked to 21 baits when it was used as a prey but no preys as a bait; on the other hand, proteins SRB4, STD1, and APG17 act similarly as bait and prey. On the basis of this observation, one could envision three basic types of protein functions in the experimental setting (Fig. 1b): pure bait (blue dot in Fig. 1b), pure prey (green dot in Fig. 1b), and both bait and prey (red dot in Fig. 1b, abbreviated as BP in this paper). These protein types can combine to form a network such as that shown in Fig. 1c. The same features exist in all other protein-protein interaction networks we analyzed, i.e., some proteins can link to a number of other proteins when used as either bait or prey, but most proteins "link better" as either a prey or bait. Figure 1d shows the percentage of the three types of proteins in several experimental datasets.

Here we first defined the sub-network composed of the proteins which have both bait and prey functions, and the links among these proteins (red dot and links in Fig. 1c), as a "core sub-network" (CSN). Although the proteins can act as both bait and prey, some of them are still very biased towards one behavior or the other, resulting in very asymmetrical bait and prey behaviors of the proteins. The pure baits and pure preys are the extreme cases of this asymmetrical bait and prey behavior. We first exclude these extreme proteins and develop later a quantitative method to further refine the CSN.

**Figure 2:**
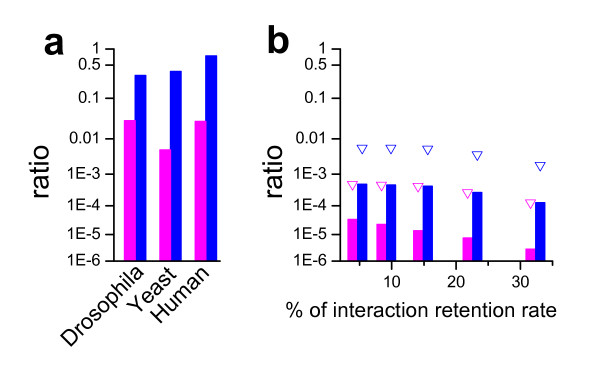
**Evidence supporting that experimental sampling of protein interaction networks is not random**. The ratio is defined as the proteins which link to 10 or more proteins when used as baits but none as preys versus either the total proteins of the network (magenta) or the total proteins who link to more than 10 proteins as bait no matter how many proteins linked to when used as preys (blue). a. Experimental datasets of yeast, Drosophila, and human. b. Truncated power-law network for different interaction retention rates. The sampling was done as follows. We first generated a large network and random sampled the proteins and interactions in a way that matches the size and number of links in the Drosophila dataset. Note that to maintain the sampled network at the size of the Drosophila dataset for different retention rates of interactions, one needs to start with networks of different original sizes. 5000 random samplings were carried out for each retention rate. Bars represent the average ratio and triangles are the maximum of the 5000 samplings.

Ideally, if the interactions (in this study, we count A–B as one link, but A → B with A as bait and B as prey and B → A with B as bait and A as prey, as two interactions) between the proteins were completely sampled, there would no pure baits or pure preys. One can attribute the occurrence of the asymmetrical properties to the limitations of experimental systems or to the proteins being artificially sorted by the way the experiments were carried out. However, the asymmetrical bait and prey properties can also occur with random sampling if the sampling of the interactions is incomplete. To exclude that the measured network is indeed a randomly sampled sub-network of the original network, we did further analyses of the experimental datasets. Firstly, if the experimental sampling were indeed random, then the number of observed "pure bait" and "pure prey" proteins following an incomplete sampling should be approximately equal; in fact, however, these numbers are quite different in the experimental datasets (Fig. 1d). Secondly, if the sampling is done randomly with incomplete sampling of interactions, the chance of experimentally detecting a protein that links to many other proteins as a bait, but to none as a prey, should be very low. This is supported by the results shown in Fig. 2, in which we calculated the ratios of the proteins which link to 10 or more proteins when used as baits but none as preys, to either the total proteins of the network (magenta) or the total proteins who link to more than 10 proteins as bait no matter how many proteins are linked to it when acting as prey (blue). In the real datasets (Fig. 2a), the ratios are very high, while they are much lower in true random sampling simulations (Fig. 2b). We calculated these ratios for simulated Erdös-Rényi random, exponential, power-law, and truncated power-law networks, and they are all in the same order of magnitude as the results for the truncated power-law network shown in Fig. 2b. The high chance (Fig. 2a) that a protein links to many proteins as a bait, but to none as a prey, indicates that the proteins were sorted into different categories (pure bait, pure prey, both bait and prey) by the experiment.

The results in Fig. 1d and Fig. 2 show that the bait and prey behaviors in experimental datasets differ substantially from a true random sampling; in other words, experimental sampling is not random. This supports the idea that bait/prey preference is an artifact of the experimental limitations and/or sampling methods, as previously suggested by Aloy and colleagues [24], and Maslov and Sneppen [25,26]. Therefore, based on the available theory on random sampling [7,8], one cannot extrapolate the topological information from the experimentally measured sub-networks to the entire network.

### Effects of experimental sampling on network topology

To show how the experimental sampling affects the topological information, we first studied effects of the ratio of the three types of nodes in the sampled network on the degree distribution and motif structure. We generated three theoretical networks (15,000 nodes each) with different topologies (Erdös-Rényi random distribution with an average connectivity equals 40, exponential distribution *p*(*k*) ∝ *e*^-0.025*k*^, and scale-free distribution *p*(*k*) ∝ *k*^-1.4^) and used the Drosophila protein-protein interaction (DPPI) network by Giot et al [20] as if it were a theoretical network without the original bait and prey information.

**Figure 3:**
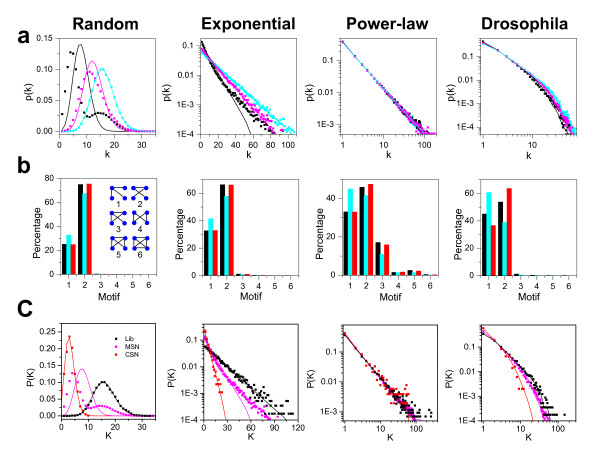
**Effects of experimental sampling on the network degree distributions and motifs**. a. Degree distributions for different theoretical networks. The libraries of 6,000 proteins were first randomly sampled from the original networks of 15,000 proteins. For the DPPI network, 5980 proteins were randomly sampled from the original 7049 proteins. To simulate the effects of experimental sampling on degree distribution (symbol), different ratios of red:blue:green (red: bait and prey, blue: pure bait, and green: pure prey as defined in Fig. 2) were then assigned in the sampled network. These ratios include red:blue:green, 1:0:0 (cyan); red:blue:green, 1:1:1 (magenta); and red:blue:green, 2:1:7 (black). For comparison, the degree distribution (line) of a randomly sampled network of the same size for each ratio from the parent network is also shown. b. The percentage of different four-node motifs for the library (black), the sampled network (magenta) of red:blue:green of 2:1:7, and the CSN (red) from the sampled network, for the same four types of networks shown in a. c. Comparison of degree distributions between sub-networks (symbols) and randomly sampled networks with the same size (lines). The library (black) is the same in a; the sampled network (magenta) has the ratio red:blue:green of 2:1:7; and the CSN (red) is from the sampled network.

To mimic the experimental sampling, we randomly selected 6000 nodes from the 15,000-node parent networks (for the DPPI network, 5980 proteins were randomly sampled from the original 7049 proteins) as the experimental libraries, and randomly assigned proteins (independent of degree/link number) in the libraries to be pure baits, pure preys, or BPs (proteins that can act as both bait and prey), with certain probabilities. Different ratios between these three types were thus obtained. We then applied the following rules to the interactions: (i) any interaction originating from a pure prey or terminating on a pure bait is forbidden (see Additional file 1 Fig. 2); (ii) all other interactions are detectable according to a probability *q *(In Fig 3, we focused on the effects of the three types of proteins but not the random sampling of the interactions, and thus we chose *q *= *1*); and (iii) that a link between protein A and protein B exists in the measured networks when at least one of interactions A → B and B → A is detected. For comparison, we also performed a true random sampling of the original networks using the same number of nodes as the simulated experimental networks. Note that in the resultant network, one observes new ratios between the pure preys, the pure baits, and the BPs, which are different from the prior assigned rations. This is because of incomplete sampling, i.e., some of the prior assigned BPs become either pure baits, pure preys, or isolated nodes (which are not detected) in the resultant network. In this study, when we refer to a protein as a pure bait, a pure prey, or a BP, we refer to the observed behavior of the protein, not the prior assigned property.

Figure 3a shows the degree distributions of the four types of networks. For Erdös-Rényi random and exponential networks, the degree distribution of the simulated experimental network (symbols) becomes increasingly different from the corresponding random sample network (lines) as the proportion of pure baits or preys increases. For the power-law network, the degree distribution is unchanged. The DPPI network exhibits a truncated power-law distribution, and therefore minor effects are observed for small connectivity since it is dominated by a power-law component, but larger effects of the differences in sampling manifest for larger connectivity due to the exponential tail of the degree distribution. The sub-network within the measured network that contains only BPs–which is a random sample of the library and therefore a random sample of the full network–may maintain the distribution characteristics of the full network. However, all links between two pure baits and between two pure preys are missing in the measurement. As such, the contribution of the pure baits and pure preys are biased and may change the characteristics of the degree distribution. An extreme example of this phenomenon can be observed with a random degree distribution with protein ratio of 2:1:7 (BP: pure bait: pure prey) in which the observed degree distribution of the sub-network displays two peaks with the smaller one contributed by the pure preys alone.

We also counted the sub-graphs of the networks as performed in previous studies [27-29]. Theoretically, a randomly sampled sub-network retaining all links (*q *= *1*) should maintain the ratios between different types of motifs, based on the following argument: a given four-node motif (for example) in the parent network remains intact in the sampled sub-network if and only if all 4 nodes are in the sub-network. If the sub-network is sampled by selecting nodes with a probability *p*, then a four-node motif survives with probability *p*^4^. Since all motifs have the same survival probability, the percentage of different motif types will not change in the randomly sampled sub-network. On the other hand, in the simulated experimental network, the three types (BP, pure bait, pure prey) may change the survival probability, i.e. the probability that the link is maintained in the sample. For example, for the two motifs: Motif 1 (A–B, A–C, A–D) and Motif 2 (A–B, A–C, B–D) (see Additional file 1 Fig. 2b), if nodes A and D are pure baits, B and C are BPs, it is impossible for Motif 1 to survive to the sampled network as the link A–D will invariably be missed. In contrast, Motif 2 has the survival probability of *p*^4^. Thus, the ratio of the three types of nodes we define in this study can determine (arbitrarily) the percentage of interaction motifs observed in the sampled network. Changes in this ratio, over which the experimenter does not have control, can alter the perceived topology and motif make-up of the network.

Figure 3b shows the percentage of six different four-node motifs for each of the four types of original networks (black bar), for the simulated experimental networks (cyan bar), and for the sub-network composed of BPs (red bar). The percentage of the motifs detected in the sub-network composed of BPs is almost unchanged from the original network (although larger variations occur in the DPPI dataset). However, the percentage of motif 1 increases and motif 2 decreases in the simulated experimental network (witness this same trend for all four types of networks). Note that although the experimental procedure has almost no effect on degree distribution if the network is scale-free, the network motifs change in the similar manner as in other types of networks.

Figure 3c shows the degree distributions of the sub-network composed of BPs (CSN) within the simulated experimental networks with protein ratio of 2:1:7 (BP: pure bait: pure prey). For all four types of networks, the distribution of CSN (red symbols) closely matches the degree distribution of the corresponding random sample network (red line). For Erdös-Rényi random and exponential networks, the degree distribution of the simulated experimental network (magenta symbols) is different from the corresponding random sample network (magenta lines). Fig 3b and Figure 3c imply that the sub-network composed of BPs (CSN) is the random sample of the full network and therefore retains the topological information of the full network. However, the simulated experimental network will change the topological information: in Erdös-Rényi random and exponential networks, the change includes both degree distribution and motif distribution; for power law and DPPI networks, the change involves the motif distribution.

### Filtering core sub-network within an experimental dataset

Based on our analysis above, it is not surprising that the bait/prey preference affects the network topology so that it cannot be used to predict the topology of the parent network. But it is also not non-intuitive that the core sub-network (CSN) which is composed of only BPs (the red dots and lines in Fig. 1c) may better reflect the topological information of the parent network since the proteins in that network are somehow less biased or better represented. It is obvious that in our computer simulated networks (Fig. 3), the CSN is a true random sample of the full network; therefore, the degree distribution and motif structure of this random sample agree very well with the original network. However, in the experimental datasets, even in the CSN as defined above (the red dots and lines in Fig. 1c), most of the proteins are not equally effective as baits and as preys, but rather, exhibit a bias behavior as either bait or prey. This feature exists in all protein-protein interaction networks we analyzed. For example, protein SRB4 in the yeast dataset (Fig. 1a) is very effective when used as a bait, but much less so as a prey. Specifically, it linked to 95 (we denote this number as m) preys when it was used as a bait. Among the 95 preys, 23 (we denote this number as m_1_) proteins were also labeled as baits in the dataset. This indicates that if SRB4 is also effective as a prey, it should (theoretically) be linked to at least these 23 proteins when it was a prey. However, it was only linked to 3 (we denote this number as n) baits (TAF17, YNR024W, and RIF2), 2 of which (we denote this number as n_1_) themselves behave as preys. Unfortunately, none of the 3 proteins that SRB4 linked to when it was a prey belonged to the list of 23 proteins that should have been able to link with SRB4. If SRB4 was equally effective as both bait and prey, it would link to the same 23 baits when it is used a prey, resulting in 23 bidirectional interactions; however, none of these bidirectional links were detected in the experiment. In fact, in all the available experimentally-measured datasets [11,12,20,23], the incidence of bidirectional links is very low. For example, in the yeast network by Ito *et al *[23], there are only 74 bidirectional interactions out of 4,549 total interactions among 3,278 proteins. In the human network by Stelzl *et al *[12], 8 out of 3,269 interactions are bidirectional. In the DPPI network by Giot *et al *[20], the value is 266 out of 20,405. Most of these bidirectional interactions (260 out of 266) were retained in their high-confidence dataset though the total interactions were reduced to 4,780, suggesting that most of the detected bidirectional interactions are true links. The reason for the prevalence of this incongruent behavior of proteins in one scenario versus another (i.e. preferential actions as bait or prey) is unclear, but may result from altered protein folding, differences in post-translational modification, necessity of tertiary interactions, or other factors.

According to our analysis above, exclusion of pure baits and pure preys does not eliminate the biased behavior of proteins from the CSN. To further refine this network, we first define two quantities–the bait score and prey score–to quantitatively characterize the experimental behavior of individual proteins. These two quantities are empirically defined as: *bait score = m/n*_1_, *prey score = n/m*_1 _(truncated to 1 if greater than 1). The rationale for these definitions is as follows. For the hypothetical Protein X, m is the number of preys to which Protein X links when it is a bait protein, among which m_1 _proteins are themselves also baits in the experiment. The number of baits to which Protein X links when it is a prey protein, is denoted by the term n. In the perfect experiment, when Protein X functions as a prey it should therefore link to at least m_1 _proteins (i.e. m_1 _should be equal to n). This of course is not the case in a real experiment, however, and therefore a protein's behavior as a prey is quantified by n/m_1_, i.e., the prey score. In the experimental setting, n can be larger than m_1_, and m_1 _= 0 for the pure preys; therefore, once n>m_1_, we set the prey score to be the maximum 1. Similar nomenclature is used to label proteins from the prey perspective. For a given Protein X, n is the number of baits to which it links when it is a prey, among which n_1 _proteins are themselves also preys in the same experiment. As with the bait score above, the experimental data does not show the idealized relationship in which all interactions are detected from both directions, and therefore the bait score is calculated as m/n_1_. Relating these two scores together in the idealized scenario for a BP protein the bait score = prey score = 1, pure baits have bait score = 1 and prey score = 0, and pure preys have bait score = 0 and prey score = 1. For the proteins in red nodes in Fig. 1c, both scores range from 0 to 1, reflecting the aforementioned point that amongst the proteins functioning as both bait and prey, there is a range over which the relative abilities of individual proteins in each of these roles is distributed.

**Figure 4:**
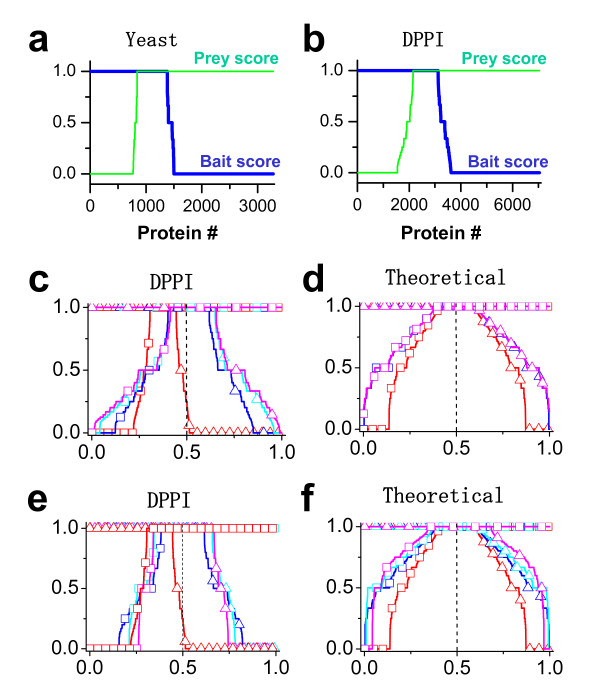
**Testing the bait and prey symmetry of the network**. a and b. bait (blue) and prey (green) score distributions for the yeast dataset [23] and the DPPI dataset [20]. c and d. bait (square) and prey (triangle) score distributions for the networks defined by the following operation: network level one (blue) defined from the original network (red) by bait score >0 and prey score >0; network level two (cyan) defined from network level one by the same criterion with the bait and prey score recalculated for the network level one; and so forth for network level three (magenta). e and f. bait (square) and prey (triangle) score distributions for CSNs defined from the same original network with different bait and prey core thresholds. Red: original network; Blue: bait score ≥ 0.2 and prey score ≥ 0.2; Cyan: bait score ≥ 0.5 and prey score ≥ 0.5; Magenta: bait score ≥ 0.8 and prey score ≥ 0.8. The theoretical network is a truncated power-law network.

Figures 4a and 4b show the two scores for the Yeast dataset and the DPPI dataset (scores for other datasets are shown in Additional file 1 Fig.S3). We can define the core sub-network (CSN) by filtering out the proteins with low bait and prey scores. This is done by selecting a real number s between 0 and 1 and all of the nodes whose bait and prey scores are ≥ s are members of the CSN. The proteins with both higher bait scores and higher prey scores are less biased and more likely to provide accurate topological information. If we filter the dataset by setting the bait score and prey score to be greater than zero, the resultant CSN looks like Fig. 1c, i.e., all the pure baits and pure preys are filtered out. This filtering step is also shown for the Drosophila network in Fig 4C. If one further redefines the bait and prey scores for the CSN, the new score distribution becomes much more symmetric (Fig. 4c). For a randomly-sampled network, the score distribution is symmetric and repeating this sampling process retains the symmetry (Fig. 4d). If we filter the dataset with different bait and prey score criteria, as the score threshold increases, so does the degree of symmetry in the sampled data (Fig. 4e and 4f). Therefore, the CSN has symmetry similar to that of the randomly sampled networks, providing strong evidence that the CSN's behavior is more akin to that of a true random sample. We calculated the same ratios as shown in Fig. 2 for the CSN of the DPPI dataset–the ratios equal to zero–which is similar to the randomly sampled networks as in Fig. 2b.

**Figure 5:**
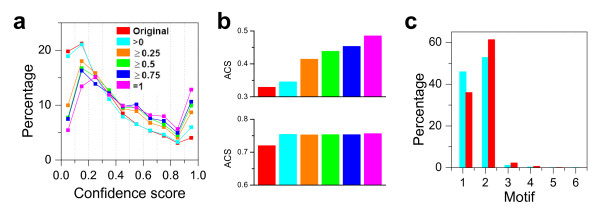
**Confidence score of core sub-network**. a. Percentage of links in each bin of confidence score for the DPPI dataset [20] (red) and for several CSNs with distinct bait and prey scores in other colors (cyan, ≥ 0; orange, ≥ 0.25; green, ≥ 0.5; blue, ≥ 0.75; and magenta, = 1). In all cases, the CSNs have more links from higher bins of confidence, an independent estimation of the reliability of the data from the experimental setting. b. The average confidence score for the full DPPI network (upper panel) and the high confidence network (lower panel) for different CSNs defined by the same range of bait and prey score thresholds as in a (coloring same as in a). c. The percentage of the four-node motifs of the DPPI dataset (cyan) and of CSN (red) defined by bait and prey score ≥ 0.5.

In the DPPI dataset by Giot et al [20], a confidence score was assigned to each link in the measured network on the basis of experimental data. Figure 5a displays the percentage of links in 10 bins of confidence score for the DPPI network (red line). The other lines are the confidence scores for different CSNs generated from the DPPI network using different bait and prey scores. Note that for all levels of confidence, the percentage of links was higher for CSN regardless of the bait and prey scores. This is particularly evident in higher bins of confidence, emphasizing that the CSN approach identifies (in an unsupervised manner) protein interactions that were experimentally assigned higher confidence. The average confidence score of the DPPI network is 0.328; however, the average confidence score of the CSN (for the highest bait and prey scores) increases to 0.485. Even for the high-confidence DPPI dataset [20], the average confidence score of the CSN is still higher than that of the whole sampled network (Fig. 5b), supporting the CSN method described in this paper as a reliable independent means to assess the topology of the entire network. Lastly, the ratio of pure baits to pure preys is much closer to 1 (which, as described above, is the ideal scenario for a true random sample) when the CSNs are examined as compared to the total experimentally measured network (Fig. 5c), indicating that the CSN may better approximate a random sample of the original network. In fact, this same feature exists in the other experimental datasets [12,23] we evaluated (data not shown), that is, the ratio of pure baits to pure preys approaches 1 for the CSN.

When the original DPPI dataset was filtered into the high-confidence one [20], the protein number collapsed from 7048 to 4679 (66% of initial value) and the link number from 20405 to 4780 (23% of initial value). For the CSN generated with bait and prey scores ≥ 0.5 before filtering with confidence score, there were 1149 proteins with 1834 links, of which 130 links were bidirectional, and the average confidence score was 0.438. After the filtering, 702 (61%) proteins, 854 (47%) links, and 126 (97%) bidirectional links remained, and the average confidence score was 0.747. This exercise demonstrates that the links in the CSN have a much higher retention rate (47% vs. 23%) when filtered with confidence, in further agreement with the higher average confidence score of interactions in the CSN. This conclusion is further substantiated if we regenerate the CSN (with the same bait and prey scores) after filtering the DPPI network to the high confidence DPPI network on the basis of the experimental data: this new CSN has 937 (602 are identical to those in the unfiltered CSN) proteins, 902 (450 identical) links, 223 bidirectional links, and an average confidence score of 0.753, which is substantially increased in comparison to when the filtering is done *after *the CSN is defined from the DPPI network. Interestingly, 84% (223/266) of the bidirectional links were retained when the CSN was defined after filtering the DPPI network to the high confidence DPPI network, versus 47% (126/266) retention of bidirectional links when defined from the DPPI network prior to confidence score filtering. Thus, this CSN approach is an independent (and complementary) method to identify high confidence links more likely to harbor accurate topological information.

We also compared the motif distributions of the DDPI dataset and their CSNs (Fig. 5c). The percentage of the Motif 1 is higher, while that of Motif 2 is lower, in the DPPI network as compared to those observed in the CSN, which agrees with the theoretical analysis in Fig. 3. This is also true for the other experimental datasets (Additional file 1 Fig.S4).

Based on the analyses above, we hypothesize that the CSN within the experimentally sampled sub-network is a closer approximation of a random sample and thus retains the topological information of the original network better than the entire experimental sample. Theoretically, filtering the experimental datasets using our method with higher bait score and prey score thresholds, one can obtain a better CSN. However, due to the limited number of proteins in the network, higher bait and prey scores result in fewer proteins in the CSN, which may cause the CSN to be too small to faithfully retain the topological information of the parent network.

### What are the degree distributions of protein-protein interaction networks?

**Figure 6:**
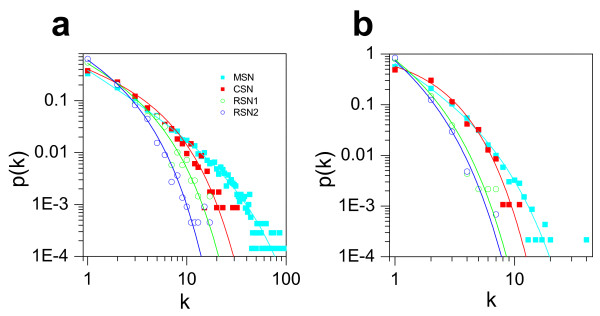
**Degree distribution of the core sub-network**. The degree distributions of the original dataset (cyan) and the CSN (red) and two randomly sampled subnetworks for the DPPI dataset (a) and the high-confidence DPPI dataset (b) by Giot et al [20]. Here CSNs are defined by bait and prey score ≥ 0.5. RSN1: randomly sampled network from MSN, in which the protein number is the same as in CSN. RSN2: randomly sampled network from MSN, in which the number of edges is the same as in the CSN. The functions for the lines in a are: *p*(*k*) = 0.4*k*^-1.2^*e*^-0.038*k *^(cyan); *p*(*k*) = 0.5*k*^-0.6^*e*^-0.22*k *^(red); *p*(*k*) = 0.7*k*^-0.85^*e*^-0.3*k *^(RSN1); *p*(*k*) = 1.0*k*^-0.85^*e*^-0.5*k *^(RSN2). The functions for the lines in b are: *p*(*k*) = 0.85*k*^-1.26^*e*^-0.27*k *^(cyan) and *p*(*k*) = 1.2*k*^-0.01^*e*^-0.75*k *^(red), *p*(*k*) = 1.9*k*^-1.0^*e*^-0.9*k *^(RSN1); *p*(*k*) = 1.9*k*^-1.2^*e*^-0.95*k *^(RSN2).

A number of studies have suggested that protein-protein interaction networks are scale-free [3,4,10-13,18], whereas other studies have contested this interpretation [19-22]. Han et al [9] showed that the scale-free nature may be due to the low sampling rate and imperfect sampling methods which can cause a sub-network from a Erdös-Rényi random network to appear scale-free. For this to happen, a key feature is the loss of the peak in the binomial distribution of the random network. Since the peak is located at [Nγ]~[(N-1)γ] = [<k>] ([x] is the integer part of x, N is the size of the original network, γ is the sampling rate, and <k> is average connectivity of the sampled sub-network, see Additional file 1 text for details), when <k><2, the peak will disappear. However, the average connectivity <k> of most of the measured networks is greater than 2, even for some of the CSNs we examined (Additional file 1 Table S1), indicating that the protein-protein interaction networks may not be random networks. On the other hand, our analysis shows that if the protein-protein interaction networks are scale-free (that is, if they have a power-law distribution), the degree distributions of either a random sample, an experimental sample or the CSN all closely resemble the same power-law distribution of the original network (see Fig. 3). This may be true even though a randomly sampled sub-network of a scale-free network may not truly be scale-free in the theoretical sense, as shown by Stumpf et al [7]. In fact, most of the experimental datasets exhibit a truncated power-law distribution *p*(*k*) ∝ *k*^-*δ *^*e*^-*εk *^(see Additional file 1 Fig.S4), and for the DPPI dataset (Fig. 6a), it is well fit by *p*(*k*) ∝ *k*^-1.2 ^*e*^-0.038*k *^as shown by Giot et al [20]. A CSN with both bait and prey scores greater than or equal to 0.5 has a degree distribution close to *p*(*k*) ∝ *k*^-0.6 ^*e*^-0.22*k*^, which has a larger exponential component but smaller power-law component than the DPPI network. For the high-confidence dataset of the DPPI network (Fig. 6b), it can be well fit by *p*(*k*) ∝ *k*^-1.26 ^*e*^-0.27*k*^, while the CSN defined by both bait and prey scores greater than or equal to 0.5 has a degree distribution *p*(*k*) ∝ *k*^-0.01 ^*e*^-0.75*k *^which is almost completely exponential. To show that this effect is not due solely to the reduction in network size, we also show the degree distributions of two random subsets of the experimentally sampled network: one where the protein number is the same as that of the CSN (called random sample 1) and the other in which the link number is the same as that of the CSN (called random sample 2), both of which have degree distributions that are very different from the CSN. In other datasets we analyzed, the degree distributions of CSNs all have a smaller power-law component and a larger exponential component as compared to the original datasets (Additional file 1 Fig.S4). However, we are not able to completely rule out that the reduction in network size contributes to the enhancement of the exponential component. The two randomly sampled networks in Fig. 6a are not very different from the CSN in both the power-law component and the exponential component. While the networks in Fig. 6b have much stronger power-law components than the CSN, there are relatively few data points making up the degree distribution for the randomly sampled networks.

## Discussion

The present study provides an improved method for extracting accurate topological information about real protein-protein interaction networks from experimentally-obtained sub-networks. The fundamental conclusions of this study can be summarized as follows: (i) random sampling of networks preserves topological information, regardless of the type of network analyzed; and (ii) experimental protein-protein interaction studies have well-established limitations that make their method of sampling non-random; however, (iii) definition of a CSN that contains proteins that behave experimentally as both baits and preys better approximates a random sample and therefore increases the accuracy of topological assessment of protein-protein interaction networks. We show that sampling of theoretical protein interaction networks with exponential, random or scale-free topology in a manner that takes into account experimental limitations, can (and indeed, usually does) produce a sample with scale-free topology; it is given that samples of protein interaction networks appear scale-free; from this, however, it cannot be concluded (as has been previously attempted) that protein interaction networks are scale-free.

Based on our method of defining CSN from the experimental datasets, we show that the degree distribution of the original network may not be scale-free, but may in fact exhibit an exponential distribution. Protein interaction analyses have unavoidable limitations including false positive and negative identifications [30-33] and assumed binary interactions, as mentioned above. We suspect that these false positives may contribute to the observed power-law component of the protein-protein interaction networks based on the following rationale: (i) the high-confidence Drosophila network (purportedly containing fewer false positives [32]) has a stronger exponential component (also verified by Przulj and colleagues [21]) and the CSN has an even higher confidence score and stronger exponential component (Fig. 5 and Figs. S4); (ii) many proteins preferentially behave as either baits or preys but not both, suggesting an experimentally-introduced preferential attachment phenomenon (introduction of hubs by experimental bias) which, as shown by Barabasi and Albert [34], is a key factor for occurrence of power-law distributions; and (iii) the degree distribution of a mammalian protein-protein interaction network obtained by Ma'ayan *et al *[29] from the literature, which should have a much lower rate of false positives, exhibits an almost purely exponential distribution (Additional file 1 Fig. S5). Additionally, the failed detection of links between certain proteins (the green ones or blue nodes in Fig. 1c) due to the aforementioned experimental considerations may contribute to the high rate of false negatives, which may thereby also contribute to the power-law component of the distribution. Although we show evidence that the degree distribution of protein-protein interaction networks might exhibit stronger exponential component, further detailed analyses are needed to substantiate this conclusion.

Determining with high confidence topological information about protein-protein interaction networks from the properties of a smaller, experimentally measured, sub-networks has been challenging [35-37]. However, the topologies of the networks are extremely important for their function and robustness [1-4,38,39].

## Conclusion

In this study, we have developed an improved method for extracting topological information for cellular protein-protein interaction networks from experimentally-obtained datasets. As structure, or network anatomy, is a necessary precursor to understanding function, or network physiology, these findings enhance our ability to use existing experimental methods for protein-protein interaction analysis to investigate the behavior of these networks *in vivo*.

## Methods

### Experimental datasets

The experimental datasets analyzed in this study were either downloaded from the related websites or kindly provided by the authors of the following references [2,9,11,12,20,23,29,31,40-42].

### Theoretical networks

Theoretical networks were generated following the method by Bender and Canfield [43], that is, we assigned a desired number of edges for each node following the theoretical distribution, then randomly linked a pair of nodes to make an edge, and decreased the link number for both nodes by one until all edges were assigned to nodes without repetition. Random networks were generated according to the Erdös-Rényi model binomial degree distribution represented by: $p(k)=C_{N-1}^{k}\gamma^{k}{\left(1-\gamma \right)}^{N-1-k}$.

### Simulated experimental networks

To mimic the experimental sampling, we first generated the theoretical parent networks with *N *nodes by the method mentioned above. Then we randomly selected *M*(*M*<*N*) nodes from the *N*-node parent network, and randomly assigned the nodes in the M-node network to be pure baits, pure preys, or both baits and preys with different probabilities independent of the number of links of the nodes. We then applied the following rules to the links of the selected nodes:

1) Any interaction starts from a pure prey or ends at a pure bait is forbidden;

2) For the allowed interactions, each has a probability *q *(in the simulations in Fig 3, we used *q *= *1*) to be detected;

3) A link A–B exists when at least one of interactions A → B and B → A is detected.

### Motif detection

We detected the motifs using the software ***mfinder1.2 ***developed by U. Alon's lab [44].

## Authors' contributions

LY carried out the computer simulations, participated in research design and drafting the manuscript. TMV participated in design and discussion of the research, and helped to draft the manuscript. ZH, WRM and JNW participated in discussion of the research. ZQ designed and directed the research, and drafted the manuscript. All authors read and approved the final manuscript.

## Supplementary Material

Additional file 1 Supplementary figures. Click here for file
