# Supplementary material for: Deducing topology of protein-protein interaction networks from experimentally measured sub-networks
Source: BMC Bioinformatics. 2008 Jul 3;9:301. doi: 10.1186/1471-2105-9-301 (PMC2474618; doi:10.1186/1471-2105-9-301)
Supplement: Additional file 1 — Supplementary figures. [file 1471-2105-9-301-S1.doc]

Deducing Topology of Protein-Protein Interaction Networks from Experimentally Measured Sub-Networks

Ling Yang, Thomas M. Vondriska, Zhangang Han, W. Robb MacLellan, James N. Weiss, Zhilin Qu

# Online Supplementary Materials

**Fig.S1.**

**Fig.S1**. **Topological characteristics of randomly sampled networks.** a. Degree distributions of randomly sampled networks from a random network (10,000 nodes and 37,470 links), an exponential network (10,000 nodes and 101,933 links, $p(k)\propto e^{-0.05k}$), a power-law network (10,000 nodes and 82,998 links, $p(k)\propto k^{-1.5}$), and the experimentally obtained DPPI network (7048 nodes and 20,405 links, $p(k)\propto k^{-1.2}e^{-0.038k}$) (Giot et al., 2003). b. Percentage of the four-node motifs for the networks sampled from a random network (6,000 nodes and 45,280 links), an exponential network (6,000 nodes and 44,096 links), a power-law network (6,000 nodes and 42,125 links), and the Drosophila network (7048 nodes and 20,405 links). Inset in the first panel shows all the four-node motifs. α is the percentage of proteins sampled from the original network and β is the probability of a link is sampled.

**Fig.S2.**

**Fig.S2**. **Illustration of detectable interactions and motifs.** To mimic the experimental sampling, we randomly assigned proteins to be pure baits (blue dots), pure preys (green dots), or BPs (red dots). (a) All types of interactions (arrows from baits to preys). Solid arrows: detectable interactions; dash arrows: undetectable interactions. (b) Example of motifs under experimental sampling. In motif 1, the link between A and D is undetectable because that the interactions from both side are undetectable. In motif 2, all links are possible to be detected.

**Fig.S3.**

**Fig.S3**. Bait score (blue line) and prey score (green line) for the PPI networks of human proteins from Stelzl et al (Stelzl et al., 2005) (a); predicted interactions of human proteins from Lehner et al (Lehner and Fraser, 2004) (b); Saccharomyces cerevisiae proteins from Uetz et al (Uetz et al., 2000) (c); yeast proteins from von Mering et al (von Mering et al., 2002) (d); DIP (Salwinski et al., 2004) (e); Metazoan C. elegans proteins from Li et al (Li et al., 2004) (f); yeast proteins from Han et al (Han et al., 2004) (g); and the high-confidence DPPI dataset (Drosophila melanogaster proteins) from Giot et al (Giot et al., 2003) (h).

**Fig.S4.**

**Fig.S4**. Degree distribution (upper panel) and percentage of four-node motifs (lower panel) of original measured sub-network (cyan) and core sub-network (red) defined by bait score $\geq$0.5 and prey score $\geq$0.5 for yeast proteins from Ito et al (Ito et al., 2001) (a); yeast proteins from Uetz-Ito-Core (Han et al., 2005) (b); human proteins from Stelzl et al (Stelzl et al., 2005)(c); yeast proteins from Han et al (Han et al., 2004)(d); DIP (Salwinski et al., 2004)(e); yeast proteins from von Mering et al (von Mering et al., 2002) (f); predicted interactions of human proteins from Lehner et al (Lehner and Fraser, 2004) (g). Since the networks in e-g are very large we were not able to calculate the motifs due to extremely long computation time. It should be noted that we do not have the specific bait and prey information for some of the datasets, such as DIP. In such case, we assume that proteins listed in the left column of the dataset as baits and the right column as preys. The lines are truncated power-law distributions with the functions for each case are: **a**. $p(k)=0.8k^{-0.42}e^{-0.48k}$ (red), $p(k)=1.9k^{-0.01}e^{-1.05k}$ (cyan); **b**. $p(k)=0.72k^{-1.1}e^{-0.28k}$ (red), $p(k)=1.5k^{-0.1}e^{-0.9k}$ (cyan); **c**. $p(k)=0.55k^{-1.2}e^{-0.125k}$ (red), $p(k)=1.45k^{-0.2}e^{-0.85k}$ (cyan); **d**. $p(k)=0.45k^{-1.0}e^{-0.12k}$ (red), $p(k)=0.55k^{-0.2}e^{-0.4k}$ (cyan); **e**. $p(k)=0.4k^{-1.2}e^{-0.042k}$ (red), $p(k)=0.43k^{-0.65}e^{-0.18k}$ (cyan); **f**. $p(k)=0.16k^{-0.85}e^{-0.012k}$ (red), $p(k)=0.19k^{-0.7}e^{-0.035k}$ (cyan). **g**. $p(k)=0.2k^{-0.9}e^{-0.015k}$ (red), $p(k)=0.3k^{-0.9}e^{-0.045k}$ (cyan).

**Fig.S5.**

**Fig.S5**. Degree distribution of the mammalian cellular network constructed from data in the experimental literature by Ma’ayan et al (Ma'ayan et al., 2005). The red line is $p(k)=0.32k^{-0.1}e^{-0.23k}$.

**Table S1.**

| PPI network | Original | CSN |
| --- | --- | --- |
| Ito et al (Yeast) (Ito et al., 2001) | 2.68 | 1.54 |
| Giot et al (Fly) (Giot et al., 2003) | 5.76 | 3.21 |
| Giot et al (Fly, High confidence) (Giot et al., 2003) | 2.01 | 1.93 |
| Stelzl et al (Human) (Stelzl et al., 2005) | 3.83 | 1.58 |
| Han et al (Yeast) (Han et al., 2004) | 3.62 | 2.57 |
| Gunsalus et al (Worm) (Gunsalus et al., 2005) | 3.92 | 3.14 |
| Ito_Core (Yeast) (Ito et al., 2001) | 1.89 | 1.41 |
| Uetz _Core (Yeast) (Han et al., 2005; Uetz et al., 2000) | 1.80 | 1.19 |
| Uetz_Ito_Core (Yeast) (Han et al., 2005) | 2.15 | 1.57 |
| Li et al (Worm) (Li et al., 2004) | 2.94 | 1.54 |
| DIP (Combined) (Salwinski et al., 2004) | 5.47 | 3.65 |
| von Mering et al (von Mering et al., 2002) | 29.46 | 12.43 |
| Lehner et al (Human)(Lehner and Fraser, 2004) | 22.83 | 9.37 |

**Table S1. Average connectivity for different protein-protein interaction networks for the original dataset and CSN defined by bait score $\geq$ 0.5 and prey score $\geq$ 0.5.**

**References:**

Giot, L., Bader, J.S., Brouwer, C., Chaudhuri, A., Kuang, B., Li, Y., Hao, Y.L., Ooi, C.E., Godwin, B., Vitols, E., Vijayadamodar, G., Pochart, P., Machineni, H., Welsh, M., Kong, Y., Zerhusen, B., Malcolm, R., Varrone, Z., Collis, A., Minto, M., Burgess, S., McDaniel, L., Stimpson, E., Spriggs, F., Williams, J., Neurath, K., Ioime, N., Agee, M., Voss, E., Furtak, K., Renzulli, R., Aanensen, N., Carrolla, S., Bickelhaupt, E., Lazovatsky, Y., DaSilva, A., Zhong, J., Stanyon, C.A., Finley, R.L., Jr., White, K.P., Braverman, M., Jarvie, T., Gold, S., Leach, M., Knight, J., Shimkets, R.A., McKenna, M.P., Chant, J. and Rothberg, J.M. (2003) A protein interaction map of Drosophila melanogaster. *Science*, **302**, 1727-1736.

Gunsalus, K.C., Ge, H., Schetter, A.J., Goldberg, D.S., Han, J.D., Hao, T., Berriz, G.F., Bertin, N., Huang, J., Chuang, L.S., Li, N., Mani, R., Hyman, A.A., Sonnichsen, B., Echeverri, C.J., Roth, F.P., Vidal, M. and Piano, F. (2005) Predictive models of molecular machines involved in Caenorhabditis elegans early embryogenesis. *Nature*, **436**, 861-865.

Han, J.D., Bertin, N., Hao, T., Goldberg, D.S., Berriz, G.F., Zhang, L.V., Dupuy, D., Walhout, A.J., Cusick, M.E., Roth, F.P. and Vidal, M. (2004) Evidence for dynamically organized modularity in the yeast protein-protein interaction network. *Nature*, **430**, 88-93.

Han, J.D., Dupuy, D., Bertin, N., Cusick, M.E. and Vidal, M. (2005) Effect of sampling on topology predictions of protein-protein interaction networks. *Nat Biotechnol*, **23**, 839-844.

Ito, T., Chiba, T., Ozawa, R., Yoshida, M., Hattori, M. and Sakaki, Y. (2001) A comprehensive two-hybrid analysis to explore the yeast protein interactome. *Proc Natl Acad Sci U S A*, **98**, 4569-4574.

Lehner, B. and Fraser, A.G. (2004) A first-draft human protein-interaction map. *Genome Biol*, **5**, R63.

Li, S., Armstrong, C.M., Bertin, N., Ge, H., Milstein, S., Boxem, M., Vidalain, P.O., Han, J.D., Chesneau, A., Hao, T., Goldberg, D.S., Li, N., Martinez, M., Rual, J.F., Lamesch, P., Xu, L., Tewari, M., Wong, S.L., Zhang, L.V., Berriz, G.F., Jacotot, L., Vaglio, P., Reboul, J., Hirozane-Kishikawa, T., Li, Q., Gabel, H.W., Elewa, A., Baumgartner, B., Rose, D.J., Yu, H., Bosak, S., Sequerra, R., Fraser, A., Mango, S.E., Saxton, W.M., Strome, S., Van Den Heuvel, S., Piano, F., Vandenhaute, J., Sardet, C., Gerstein, M., Doucette-Stamm, L., Gunsalus, K.C., Harper, J.W., Cusick, M.E., Roth, F.P., Hill, D.E. and Vidal, M. (2004) A map of the interactome network of the metazoan C. elegans. *Science*, **303**, 540-543.

Ma'ayan, A., Jenkins, S.L., Neves, S., Hasseldine, A., Grace, E., Dubin-Thaler, B., Eungdamrong, N.J., Weng, G., Ram, P.T., Rice, J.J., Kershenbaum, A., Stolovitzky, G.A., Blitzer, R.D. and Iyengar, R. (2005) Formation of regulatory patterns during signal propagation in a Mammalian cellular network. *Science*, **309**, 1078-1083.

Salwinski, L., Miller, C.S., Smith, A.J., Pettit, F.K., Bowie, J.U. and Eisenberg, D. (2004) The Database of Interacting Proteins: 2004 update. *Nucleic Acids Res*, **32**, D449-451.

Stelzl, U., Worm, U., Lalowski, M., Haenig, C., Brembeck, F.H., Goehler, H., Stroedicke, M., Zenkner, M., Schoenherr, A., Koeppen, S., Timm, J., Mintzlaff, S., Abraham, C., Bock, N., Kietzmann, S., Goedde, A., Toksoz, E., Droege, A., Krobitsch, S., Korn, B., Birchmeier, W., Lehrach, H. and Wanker, E.E. (2005) A human protein-protein interaction network: a resource for annotating the proteome. *Cell*, **122**, 957-968.

Uetz, P., Giot, L., Cagney, G., Mansfield, T.A., Judson, R.S., Knight, J.R., Lockshon, D., Narayan, V., Srinivasan, M., Pochart, P., Qureshi-Emili, A., Li, Y., Godwin, B., Conover, D., Kalbfleisch, T., Vijayadamodar, G., Yang, M., Johnston, M., Fields, S. and Rothberg, J.M. (2000) A comprehensive analysis of protein-protein interactions in Saccharomyces cerevisiae. *Nature*, **403**, 623-627.

von Mering, C., Krause, R., Snel, B., Cornell, M., Oliver, S.G., Fields, S. and Bork, P. (2002) Comparative assessment of large-scale data sets of protein-protein interactions. *Nature*, **417**, 399-403.
